# Supplementary material for: A comprehensive assessment of current methods for measuring metacognition
Source: Nat Commun. 2025 Jan 15;16:701. doi: 10.1038/s41467-025-56117-0 (PMC11735976; doi:10.1038/s41467-025-56117-0)
Supplement: Supplementary file 1 — Supplementary Information [file 41467_2025_56117_MOESM1_ESM.pdf]

SUPPLEMENTARY INFORMATION

Title: A comprehensive assessment of current methods for measuring metacognition

Author: Dobromir Rahnev

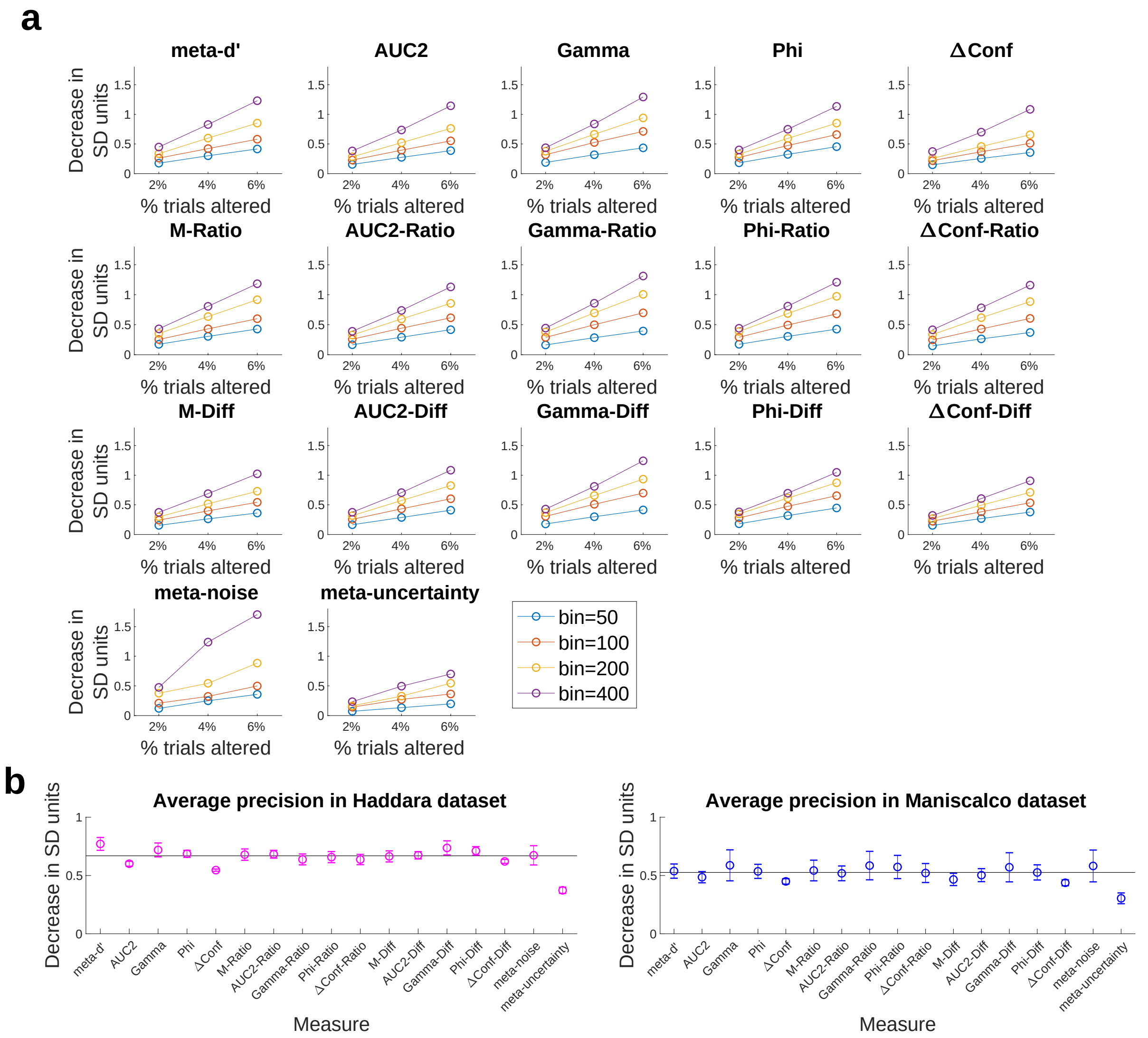

Supplementary Fig. 1. Validity and precision of each measure. (a) Validity and precision in the Maniscalco dataset. This panel is equivalent to Figure 1a (which shows detailed results for the Haddara dataset). The panel shows results of an artificial corruption of the confidence ratings where confidence for correct trials was decreased by 1, and confidence for incorrect trials was increased by 1. Each one of the 17 measures of metacognition showed a decrease with this manipulation. The plot shows the decrease in units of the standard deviation (SD) of the measure's fluctuations across different bins. The decrease was computed for bin sizes of 50, 100, 200, and 400 trials, as well as for 2, 4, and 6% of trials being corrupted. (b) Average precision in SD units for each measure in the Haddara (n = 70) and Maniscalco (n = 22) datasets averaged across the four bin sizes and the three levels of corruption. The horizontal lines represent the average value of the first 16 measures and are added to make it easier to see any differences between those measures. Error bars show SEM.

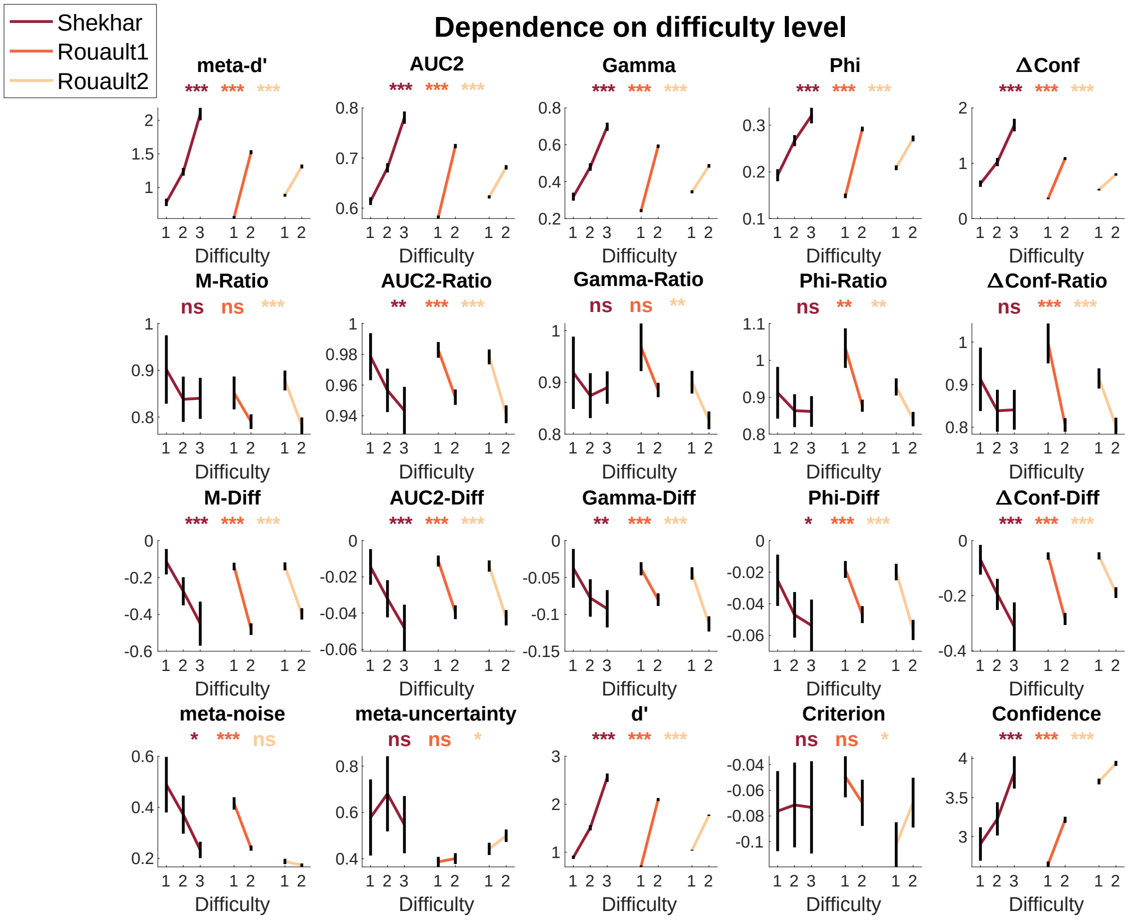

Supplementary Fig. 2. Dependence of estimated metacognitive scores on difficulty level. Estimated metacognitive ability for all 17 measures, as well as d', criterion, and confidence for different difficulty levels in the Shekhar (n = 20), Rouault1 (n = 466), and Rouault2 (n = 484) datasets. Traditional measures of metacognition (top row) all showed a strong positive relationship with task performance, whereas all Diff measures (third row) show a strong negative relationship. Ratio measures (second row) and the two model-based measures (*meta-noise* and *meta-uncertainty*) performed much better but still showed weak relationships with task performance. Note that larger numbers on the x axis indicate easier conditions. ***, $p < 0.001$; **, $p < 0.01$; *, $p < 0.05$; ns, not significant.

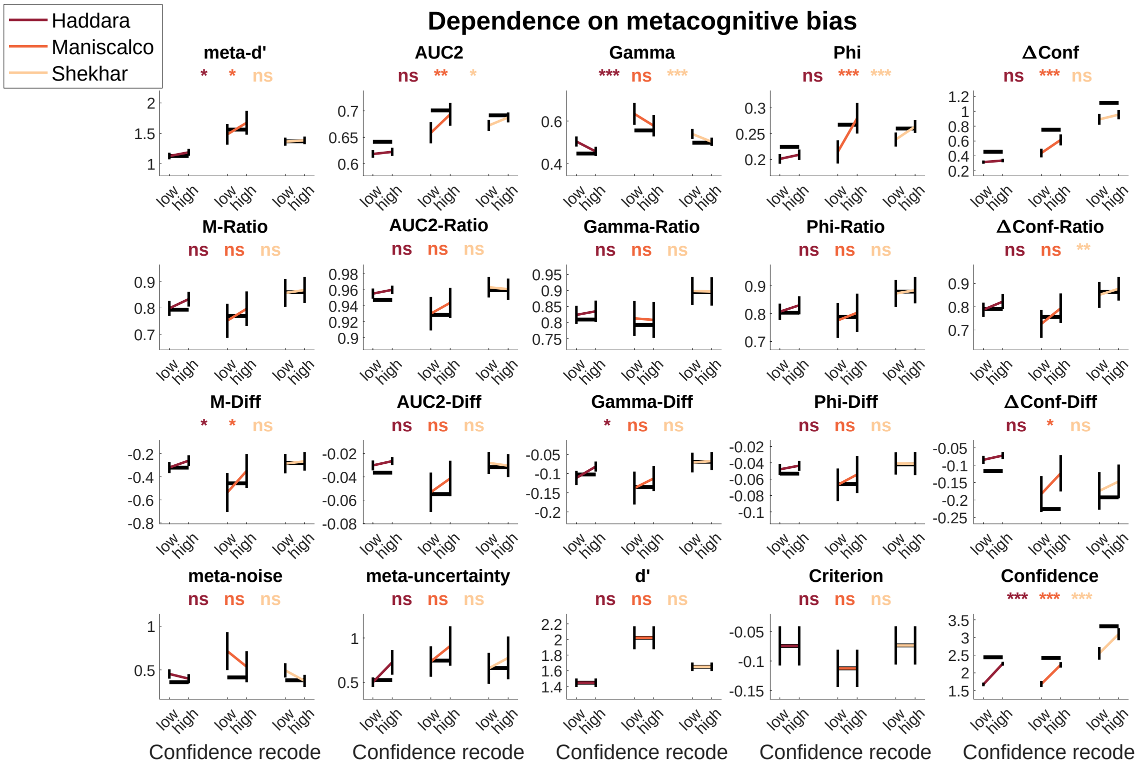

Supplementary Fig. 3. Comparison of metacognitive scores before vs. after recoding using the Xue et al. method. The figure shows the same data as Figure 3a from the main paper that plots metacognitive scores after recoding with the Xue et al. method using the Haddara (n = 70), Maniscalco (n = 22), and Shekhar (n = 20) datasets. Critically, it also displays (in thick black lines) the raw metacognitive scores before recoding using the Xue et al. method. As can be seen in the figure, the recoding did not have a strong effect on metacognitive scores relative to the metacognitive scores before recoding. Across all subjects and all 17 measures, on average 60% (Haddara dataset), 55% (Maniscalco dataset), and 52% (Shekhar dataset) of metacognitive scores were higher after recoding compared to the original scores before recoding. Thus, the metacognitive scores after recoding appear to be slightly higher on average, but this effect is quite small. Future research should address what factors may have led to this effect. Error bars show SEM. ***, p < 0.001; **, p < 0.01; *, p < 0.05; ns, not significant.

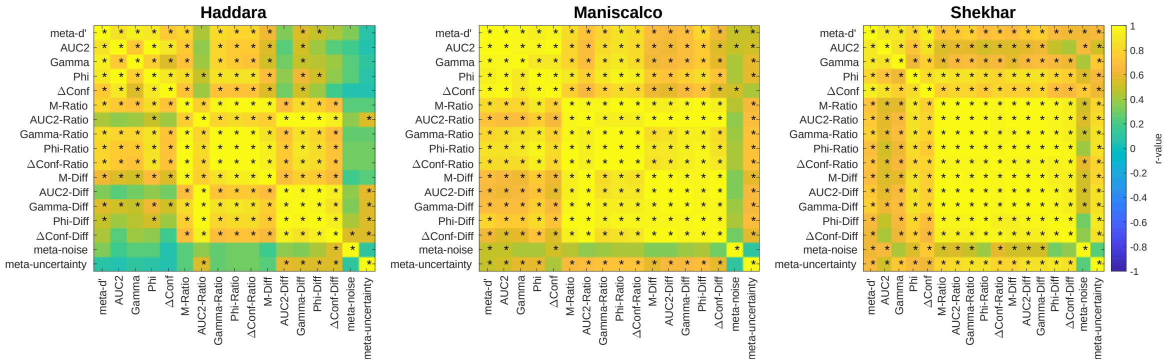

Supplementary Fig. 4. Across-subject correlations between different measures. The figure depicts r-values obtained from conducting across-subject Pearson correlations between all pairs of measures. The analyses were conducted separately for the Haddara (n = 70), Maniscalco (n = 22), and Shekhar (n = 20) datasets. Star symbols indicate significant correlation at the $p < 0.05$ uncorrected level.

Supplementary Table 1. Requirements for measures of metacognitive ability.

Requirement	Justification
High precision	Manipulations affecting metacognition should have a large effect on the measure of metacognitive ability relative to its normal fluctuations.
Independence from task performance	Giving participants an easier or more difficult task should not affect the measured metacognitive ability.
Independence from metacognitive bias	Metacognitive bias (tendency to give high or low confidence ratings) is under participants' strategic control. Whether they choose to use the lower or higher ends of the confidence scale should not affect the measured metacognitive ability.
Independence from response bias	Response bias (tendency to choose one category more than the other) is under participants' strategic control. Whether they choose to select one stimulus more than the other should not affect the measured metacognitive ability.
High reliability	For studies of individual differences, it is critical that the measure of metacognitive ability has high reliability (e.g., split-half and test-retest reliability).

Supplementary Table 2. Nuisance variables that can confound measures of metacognitive ability.

Measure	Interpretation
sensitivity (d')	Task performance computed using SDT assumptions
criterion (*c*)	Response bias computed using SDT assumptions)
confidence	Average confidence (measure of metacognitive bias

Supplementary Table 3. Dependence of estimated metacognitive scores on task performance for the Shekhar dataset. Statistical results are based on uncorrected two-sided t-tests comparing the highest to lowest difficulty level for each measure. df, degrees of freedom; CI, 95% confidence interval.

Measure	t	df	p	Cohen's d	CI lower	CI upper
meta-d'	22.616	19	<0.001	5.057	1.195	1.439
AUC2	20.612	19	<0.001	4.609	0.149	0.183
Gamma	29.238	19	<0.001	6.538	0.352	0.406
Phi	10.898	19	<0.001	2.437	0.103	0.152
DeltaConf	14.834	19	<0.001	3.317	0.910	1.209
M-Ratio	-1.240	19	0.230	-0.277	-0.166	0.042
AUC2-Ratio	-3.215	19	0.005	-0.719	-0.058	-0.012
Gamma-Ratio	-0.574	19	0.573	-0.128	-0.133	0.076
Phi-Ratio	-1.014	19	0.323	-0.227	-0.155	0.054
DeltaConf-Ratio	-1.449	19	0.164	-0.324	-0.175	0.032
M-Diff	-4.087	19	0.001	-0.914	-0.507	-0.164
AUC2-Diff	-4.006	19	0.001	-0.896	-0.051	-0.016
Gamma-Diff	-3.198	19	0.005	-0.715	-0.091	-0.019
Phi-Diff	-2.136	19	0.046	-0.478	-0.057	-0.001
DeltaConf-Diff	-3.967	19	0.001	-0.887	-0.371	-0.115
meta-noise	-2.191	18	0.042	-0.503	-0.395	-0.009
meta-uncertainty	0.229	18	0.821	0.053	-0.182	0.227
d'	23.777	19	<0.001	5.317	1.513	1.805
Criterion	0.166	19	0.870	0.037	-0.034	0.040
Confidence	14.543	19	<0.001	3.252	0.785	1.049

Supplementary Table 4. Dependence of estimated metacognitive scores on task performance for the Rouault1 dataset. Statistical results are based on uncorrected two-sided t-tests comparing the higher to lower difficulty levels (obtained using median split) for each measure. df, degrees of freedom; CI, 95% confidence interval.

Measure	t	df	p	Cohen's d	CI lower	CI upper
meta-d'	35.285	454	<0.001	1.654	0.921	1.030
AUC2	35.405	463	<0.001	1.644	0.134	0.149
Gamma	36.801	459	<0.001	1.716	0.330	0.367
Phi	24.759	460	<0.001	1.153	0.132	0.154
DeltaConf	31.666	458	<0.001	1.478	0.673	0.762
M-Ratio	-1.646	439	0.101	-0.078	-0.133	0.012
AUC2-Ratio	-4.604	462	<0.001	-0.214	-0.043	-0.017
Gamma-Ratio	-1.659	448	0.098	-0.078	-0.167	0.014
Phi-Ratio	-2.980	455	0.003	-0.140	-0.268	-0.055
DeltaConf-Ratio	-3.964	450	<0.001	-0.187	-0.281	-0.095
M-Diff	-9.809	461	<0.001	-0.456	-0.409	-0.272
AUC2-Diff	-6.327	460	<0.001	-0.295	-0.037	-0.019
Gamma-Diff	-3.931	459	<0.001	-0.183	-0.067	-0.022
Phi-Diff	-4.098	462	<0.001	-0.190	-0.042	-0.015
DeltaConf-Diff	-9.694	457	<0.001	-0.453	-0.278	-0.185
meta-noise	-6.428	445	<0.001	-0.304	-0.220	-0.117
meta-uncertainty	0.584	448	0.559	0.028	-0.042	0.078
d'	49.278	463	<0.001	2.288	1.334	1.445
Criterion	-1.659	463	0.098	-0.077	-0.041	0.003
Confidence	32.390	462	<0.001	1.505	0.521	0.588

Supplementary Table 5. Dependence of estimated metacognitive scores on task performance for the Rouault2 dataset. Statistical results are based on uncorrected two-sided t-tests comparing the higher to lower difficulty levels (obtained using median split) for each measure. df, degrees of freedom; CI, 95% confidence interval.

Measure	t	df	p	Cohen's d	CI lower	CI upper
meta-d'	15.304	476	<0.001	0.701	0.374	0.484
AUC2	13.657	480	<0.001	0.623	0.049	0.066
Gamma	13.002	476	<0.001	0.595	0.118	0.160
Phi	9.237	479	<0.001	0.422	0.049	0.075
DeltaConf	13.692	475	<0.001	0.628	0.233	0.312
M-Ratio	-3.993	479	<0.001	-0.182	-0.144	-0.049
AUC2-Ratio	-5.514	480	<0.001	-0.251	-0.050	-0.024
Gamma-Ratio	-3.033	480	0.003	-0.138	-0.125	-0.027
Phi-Ratio	-3.200	481	0.001	-0.146	-0.141	-0.034
DeltaConf-Ratio	-4.033	481	<0.001	-0.184	-0.164	-0.056
M-Diff	-7.831	476	<0.001	-0.359	-0.324	-0.194
AUC2-Diff	-6.019	478	<0.001	-0.275	-0.038	-0.019
Gamma-Diff	-5.811	475	<0.001	-0.266	-0.091	-0.045
Phi-Diff	-4.917	480	<0.001	-0.224	-0.051	-0.022
DeltaConf-Diff	-6.466	470	<0.001	-0.298	-0.184	-0.098
meta-noise	-1.120	468	0.263	-0.052	-0.035	0.010
meta-uncertainty	2.001	460	0.046	0.093	0.001	0.124
d'	48.583	469	<0.001	2.241	0.696	0.755
Criterion	2.407	482	0.016	0.110	0.006	0.056
Confidence	15.334	476	<0.001	0.702	0.191	0.247

Supplementary Table 6. Dependence of estimated metacognitive scores on metacognitive bias for the Haddara dataset. Statistical results are based on uncorrected two-sided t-tests comparing recodings biased towards lower or higher confidence ratings using the Xue et al. (2021) method. Note that the table doesn't include results for d' and c because the Xue et al. method doesn't affect the responses and thus has no effect on performance or criterion measures. df, degrees of freedom; CI, 95% confidence interval.

Measure	t	df	p	Cohen's d	CI lower	CI upper
meta-d'	2.584	69	0.012	0.309	0.014	0.107
AUC2	0.688	69	0.494	0.082	-0.008	0.016
Gamma	-4.331	69	<0.001	-0.518	-0.068	-0.025
Phi	1.257	69	0.213	0.150	-0.005	0.021
DeltaConf	1.034	69	0.305	0.124	-0.019	0.059
M-Ratio	1.994	69	0.050	0.238	0.000	0.070
AUC2-Ratio	1.130	69	0.262	0.135	-0.004	0.013
Gamma-Ratio	0.592	69	0.555	0.071	-0.027	0.049
Phi-Ratio	1.074	69	0.287	0.128	-0.020	0.065
DeltaConf-Ratio	1.693	69	0.095	0.202	-0.006	0.076
M-Diff	2.577	69	0.012	0.308	0.014	0.107
AUC2-Diff	1.202	69	0.233	0.144	-0.002	0.010
Gamma-Diff	2.334	69	0.022	0.279	0.004	0.055
Phi-Diff	0.996	69	0.323	0.119	-0.004	0.013
DeltaConf-Diff	1.383	69	0.171	0.165	-0.005	0.030
meta-noise	-1.072	69	0.287	-0.128	-0.155	0.047
meta-uncertainty	1.956	69	0.055	0.234	-0.005	0.454
Confidence	24.538	69	<0.001	2.933	0.538	0.633

Supplementary Table 7. Dependence of estimated metacognitive scores on metacognitive bias for the Maniscalco dataset. Statistical results are based on uncorrected two-sided t-tests comparing recodings biased towards lower or higher confidence ratings using the Xue et al. (2021) method. Note that the table doesn't include results for d' and c because the Xue et al. method doesn't affect the responses and thus has no effect on performance or criterion measures. df, degrees of freedom; CI, 95% confidence interval.

Measure	t	df	p	Cohen's d	CI lower	CI upper
meta-d'	2.711	21	0.013	0.578	0.044	0.336
AUC2	3.794	21	0.001	0.809	0.016	0.054
Gamma	-1.646	21	0.115	-0.351	-0.126	0.015
Phi	5.262	21	<0.001	1.122	0.040	0.091
DeltaConf	5.242	21	<0.001	1.118	0.107	0.247
M-Ratio	1.122	21	0.275	0.239	-0.039	0.129
AUC2-Ratio	1.503	21	0.148	0.320	-0.005	0.032
Gamma-Ratio	-0.129	21	0.898	-0.028	-0.081	0.072
Phi-Ratio	0.694	21	0.495	0.148	-0.055	0.110
DeltaConf-Ratio	1.841	21	0.080	0.393	-0.009	0.143
M-Diff	2.677	21	0.014	0.571	0.042	0.332
AUC2-Diff	1.681	21	0.107	0.358	-0.003	0.026
Gamma-Diff	1.098	21	0.285	0.234	-0.023	0.073
Phi-Diff	1.096	21	0.285	0.234	-0.011	0.036
DeltaConf-Diff	2.149	21	0.043	0.458	0.002	0.117
meta-noise	-0.619	21	0.543	-0.132	-0.780	0.422
meta-uncertainty	1.095	21	0.286	0.233	-0.157	0.506
Confidence	17.328	21	<0.001	3.694	0.476	0.606

Supplementary Table 8. Dependence of estimated metacognitive scores on metacognitive bias for the Shekhar dataset. Statistical results are based on uncorrected two-sided t-tests comparing recodings biased towards lower or higher confidence ratings using the Xue et al. (2021) method. Note that the table doesn’t include results for d’ and c because the Xue et al. method doesn’t affect the responses and thus has no effect on performance or criterion measures. df, degrees of freedom; CI, 95% confidence interval.

Measure	t	df	p	Cohen's d	CI lower	CI upper
meta-d'	1.988	19	0.061	0.444	-0.001	0.043
AUC2	2.804	19	0.011	0.627	0.004	0.027
Gamma	-4.284	19	<0.001	-0.958	-0.055	-0.019
Phi	5.133	19	<0.001	1.148	0.015	0.035
DeltaConf	1.747	19	0.097	0.391	-0.013	0.140
M-Ratio	1.500	19	0.150	0.335	-0.004	0.026
AUC2-Ratio	-0.830	19	0.417	-0.186	-0.009	0.004
Gamma-Ratio	-0.183	19	0.857	-0.041	-0.017	0.014
Phi-Ratio	1.898	19	0.073	0.424	-0.001	0.025
DeltaConf-Ratio	2.992	19	0.007	0.669	0.008	0.045
M-Diff	1.857	19	0.079	0.415	-0.002	0.042
AUC2-Diff	-0.844	19	0.409	-0.189	-0.008	0.004
Gamma-Diff	0.970	19	0.344	0.217	-0.004	0.012
Phi-Diff	-0.064	19	0.950	-0.014	-0.007	0.007
DeltaConf-Diff	1.767	19	0.093	0.395	-0.005	0.059
meta-noise	-1.644	19	0.117	-0.368	-0.275	0.033
meta-uncertainty	1.548	19	0.138	0.346	-0.041	0.275
Confidence	13.845	19	<0.001	3.096	0.461	0.625

Supplementary Table 9. Dependence of estimated metacognitive scores on response bias for the Locke dataset. The Locke dataset contains seven conditions with different response biases. The table reports the effect of condition in repeated measures ANOVAs. No effect is significant, except the effect of condition on criterion. η^2_p, partial eta squared.

Measure	F(6,54)	p	η^2_p
meta-d'	1.472	0.205	0.141
AUC2	0.742	0.618	0.076
Gamma	0.863	0.528	0.087
Phi	0.927	0.483	0.093
DeltaConf	0.742	0.618	0.076
M-Ratio	1.090	0.380	0.108
AUC2-Ratio	0.622	0.712	0.065
Gamma-Ratio	0.907	0.497	0.092
Phi-Ratio	0.962	0.460	0.097
DeltaConf-Ratio	0.858	0.532	0.087
M-Diff	0.911	0.494	0.092
AUC2-Diff	0.588	0.738	0.061
Gamma-Diff	0.557	0.763	0.058
Phi-Diff	0.643	0.695	0.067
DeltaConf-Diff	0.588	0.738	0.061
meta-noise	1.701	0.138	0.159
meta-uncertainty	1.570	0.174	0.149
d'	1.287	0.279	0.125
Criterion	12.185	<0.001	0.575
Confidence	0.482	0.819	0.051
