# Supplementary material for: A comprehensive assessment of current methods for measuring metacognition
Source: Nat Commun. 2025 Jan 15;16:701. doi: 10.1038/s41467-025-56117-0 (PMC11735976; doi:10.1038/s41467-025-56117-0)
Supplement: Supplementary file 2 — Reporting Summary [file 41467_2025_56117_MOESM2_ESM.pdf]

nature portfolio

Corresponding author(s): Dobromir Rahnev

Last updated by author(s): Aug 4, 2023

Reporting Summary

Nature Portfolio wishes to improve the reproducibility of the work that we publish. This form provides structure for consistency and transparency in reporting. For further information on Nature Portfolio policies, see our Editorial Policies and the Editorial Policy Checklist.

Statistics

For all statistical analyses, confirm that the following items are present in the figure legend, table legend, main text, or Methods section.

n/a	Confirmed	
☐	☒	The exact sample size (*n*) for each experimental group/condition, given as a discrete number and unit of measurement
☐	☒	A statement on whether measurements were taken from distinct samples or whether the same sample was measured repeatedly
☐	☒	The statistical test(s) used AND whether they are one- or two-sided<br>*Only common tests should be described solely by name; describe more complex techniques in the Methods section.*
☐	☒	A description of all covariates tested
☐	☒	A description of any assumptions or corrections, such as tests of normality and adjustment for multiple comparisons
☐	☒	A full description of the statistical parameters including central tendency (e.g. means) or other basic estimates (e.g. regression coefficient) AND variation (e.g. standard deviation) or associated estimates of uncertainty (e.g. confidence intervals)
☐	☒	For null hypothesis testing, the test statistic (e.g. *F*, *t*, *r*) with confidence intervals, effect sizes, degrees of freedom and *P* value noted<br>*Give P values as exact values whenever suitable.*
☐	☒	For Bayesian analysis, information on the choice of priors and Markov chain Monte Carlo settings
☒	☐	For hierarchical and complex designs, identification of the appropriate level for tests and full reporting of outcomes
☐	☒	Estimates of effect sizes (e.g. Cohen's *d*, Pearson's *r*), indicating how they were calculated

Our web collection on statistics for biologists contains articles on many of the points above.

Software and code

Policy information about availability of computer code

Data collection	Only open source data were used. No data collection was performed for the current project.
Data analysis	Analyses were performed using MATLAB 2024a (MathWorks).

For manuscripts utilizing custom algorithms or software that are central to the research but not yet described in published literature, software must be made available to editors and reviewers. We strongly encourage code deposition in a community repository (e.g. GitHub). See the Nature Portfolio guidelines for submitting code & software for further information.

Data

Policy information about availability of data

All manuscripts must include a data availability statement. This statement should provide the following information, where applicable:

- Accession codes, unique identifiers, or web links for publicly available datasets
- A description of any restrictions on data availability
- For clinical datasets or third party data, please ensure that the statement adheres to our policy

Raw data for all six experiments was obtained from the Confidence Database (https://osf.io/s46pr/). Processed data files are available at https://osf.io/y5w2d/.

Research involving human participants, their data, or biological material

Policy information about studies with human participants or human data. See also policy information about sex, gender (identity/presentation), and sexual orientation and race, ethnicity and racism.

Reporting on sex and gender	Sex or gender were not analyzed as they were not expected to be significant mediators of the effects examined here.
Reporting on race, ethnicity, or other socially relevant groupings	Race and ethnicity were not analyzed as they were not expected to be significant mediators of the effects examined here.
Population characteristics	Population characteristics are described in the original papers that collected the data and were not examined here.
Recruitment	Recruitment for each dataset is described in the original papers that collected the data.
Ethics oversight	The current study complies with all relevant ethical regulations. All analyses were performed on deidentified data from publicly available datasets and thus were exempt from Internal Review Board review.

Note that full information on the approval of the study protocol must also be provided in the manuscript.

Field-specific reporting

Please select the one below that is the best fit for your research. If you are not sure, read the appropriate sections before making your selection.

☐ Life sciences ☒ Behavioural & social sciences ☐ Ecological, evolutionary & environmental sciences

For a reference copy of the document with all sections, see nature.com/documents/nr-reporting-summary-flat.pdf

Behavioural & social sciences study design

All studies must disclose on these points even when the disclosure is negative.

Study description	The study uses quantitative data from human subjects completing different perceptual decision-making tasks and providing confidence ratings on a trial-by-trial basis. The data were downloaded from the publicly available Confidence Database (https://osf.io/s46pr). The datasets used were: Haddara_2022_Expt2, Locke_2020, Maniscalco_2017_expt1, Rouault_2018_Expt1, Rouault_2018_Expt2, and Shekhar_2021.
Research sample	Sample characteristics are described in the original papers that reported on each dataset. All datasets used data from both genders using convenience sampling.
Sampling strategy	Sampling strategies for each dataset are described in the original papers that reported on each dataset. The data from the Haddara_2022_Expt2, Rouault_2018_Expt1, and Rouault_2018_Expt2 datasets were collected online, whereas the data from the Locke_2020, Maniscalco_2017_expt1, and Shekhar_2021 datasets were collected in-lab.
Data collection	Data collection details for each dataset are described in the original papers that reported on each dataset.
Timing	Timing of data collection for each dataset is described in the original papers that reported on each dataset. Data collection periods were as follows (as reported in the Confidence Database). Haddara_2022_Expt2: 05/2020 - 07/2020; Locke_2020: 10/2017 - 04/2018, Maniscalco_2017_expt1: information not provided, Rouault_2018_Expt1: 2015-2016, Rouault_2018_Expt2: 2015-2016, and Shekhar_2021: 10/2017 - 04/2018. None of the datasets authors reported gaps in data collection.
Data exclusions	Data exclusions are described in detail in the Methods section of the paper. Overall, 58 out of 1,091 subjects were excluded (5.32% exclusion rate). The exclusion criteria were not preregistered. However, the same exclusion criteria were used in prior papers from our lab.
Non-participation	None of the papers reporting the datasets used here reported any participants declining participation.
Randomization	Participants were not randomly allocated into different groups in any of the datasets examined here. All participants completed the same experimental procedures in each dataset. The only exception was the Haddara_2022_Expt2 datasets where participants were randomly assigned to a Feedback and a No-Feedback groups. These groups were combined for the purposes of the analyses performed here.

Reporting for specific materials, systems and methods

We require information from authors about some types of materials, experimental systems and methods used in many studies. Here, indicate whether each material, system or method listed is relevant to your study. If you are not sure if a list item applies to your research, read the appropriate section before selecting a response.

Materials & experimental systems

n/a	Involved in the study
☒	☐ Antibodies
☒	☐ Eukaryotic cell lines
☒	☐ Palaeontology and archaeology
☒	☐ Animals and other organisms
☒	☐ Clinical data
☒	☐ Dual use research of concern
☒	☐ Plants

Methods

n/a	Involved in the study
☒	☐ ChIP-seq
☒	☐ Flow cytometry
☒	☐ MRI-based neuroimaging
